# Supplementary material for: Mechanisms governing the pioneering and redistribution capabilities of the non-classical pioneer PU.1
Source: Nat Commun. 2020 Jan 21;11:402. doi: 10.1038/s41467-019-13960-2 (PMC6972792; doi:10.1038/s41467-019-13960-2)
Supplement: Supplementary file 7 — Source data [file 41467_2019_13960_MOESM7_ESM.zip › Source_Data/FigureS2/FigureS2B_MotifScanOutput/KmeansCluster5/homerResults/motif11.similar.html]

motif11

## Information for motif11

C
G
T
A
A
T
C
G
A
C
G
T
C
A
T
G
C
G
T
A
C
A
T
G
A
C
G
T
G
C
A
T
A
G
T
C
C
G
A
T
A
G
T
C
T
C
G
A
  
Reverse Opposite:  

A
G
C
T
C
T
A
G
C
G
T
A
C
T
A
G
C
G
T
A
C
G
T
A
G
A
T
C
C
G
A
T
G
T
A
C
C
G
T
A
T
A
G
C
C
G
A
T
  

|  |  |
| --- | --- |
| p-value: | 1e-76 |
| log p-value: | -1.770e+02 |
| Information Content per bp: | 1.884 |
| Number of Target Sequences with motif | 136.0 |
| Percentage of Target Sequences with motif | 3.42% |
| Number of Background Sequences with motif | 115.3 |
| Percentage of Background Sequences with motif | 0.27% |
| Average Position of motif in Targets | 100.9 +/- 53.2bp |
| Average Position of motif in Background | 112.2 +/- 74.1bp |
| Strand Bias (log2 ratio + to - strand density) | -0.4 |
| Multiplicity (# of sites on avg that occur together) | 1.00 |
| Motif File: | file (matrix) reverse opposite |

### Similar de novo motifs found

|  |  |  |  |  |  |  |  |
| --- | --- | --- | --- | --- | --- | --- | --- |
| Rank | Match Score | Redundant Motif | P-value | log P-value | % of Targets | % of Background | Motif file |
| 1 | 0.962 | T G C A A T C G A C G T C A T G C G T A C T A G G C A T A G C T G T A C A G C T A G T C | 1e-76 | -176.852778 | 3.44% | 0.28% | motif file (matrix) |
| 2 | 0.917 | C A G T T C A G C T G A T C A G G C T A C T G A T G A C G C A T A G T C G C T A A G T C G C A T A T G C G C T A | 1e-72 | -168.020563 | 4.80% | 0.75% | motif file (matrix) |
| 3 | 0.916 | G T C A A T C G A G C T A C T G C G T A A C T G A C G T A C G T A G T C A C G T | 1e-68 | -157.738145 | 3.17% | 0.27% | motif file (matrix) |
| 4 | 0.950 | C G A T T C A G C G T A C T A G T C G A T G C A T A G C G A C T G T A C G T C A A G T C G C A T A T G C | 1e-64 | -148.775709 | 3.29% | 0.34% | motif file (matrix) |
| 5 | 0.860 | C T A G C T G A C T A G G C T A C G T A G A T C A G C T A T G C C G T A T A G C A C G T A G T C C G T A A G T C | 1e-58 | -135.768730 | 2.46% | 0.17% | motif file (matrix) |
| 6 | 0.781 | T A C G C T G A C A T G C T G A C G T A T A G C G C A T A T G C C G T A A T G C G C A T A T G C C G T A | 1e-56 | -129.362447 | 4.77% | 1.01% | motif file (matrix) |
| 7 | 0.805 | A C T G G T C A A T C G C G T A C G A T T A G C A C G T A G T C T G C A | 1e-36 | -83.103397 | 3.64% | 0.92% | motif file (matrix) |
| 8 | 0.764 | A C T G A G C T A C T G C G T A A C T G A C G T A C G T | 1e-21 | -50.237402 | 6.28% | 3.08% | motif file (matrix) |
